# Q-switched Nd-YAG laser alone and in combination with innovative hyaluronic acid gels improve keratinocytes wound healing in vitro

**DOI:** 10.1007/s10103-020-03145-5

**Published:** 2020-09-26

**Authors:** Anna de Filippis, Antonella D’Agostino, Anna Virginia Adriana Pirozzi, Maria Antonietta Tufano, Chiara Schiraldi, Adone Baroni

**Affiliations:** 1 Department of Experimental Medicine, Section of Microbiology and Clinical Microbioloy, University of Campania “Luigi Vanvitelli”, Naples, Italy; 2 Department of Mental Health and Physics and Preventive Medicine, Section of Dermatology, University of Campania Luigi Vanvitelli, Naples, Italy; 3 Department of Experimental Medicine, Section of Biotechnology, Medical Histology and Molecular Biology, University of Campania Luigi Vanvitelli, via De Crecchio n°7, 80138 Naples, Italy

**Keywords:** Q-switched Nd-YAG laser, Hyaluronic acid gels, Wound healing, Molecular biomarkers, Hybrid cooperative complexes

## Abstract

During the last years, several attempts have been accomplished to improve the wound healing. Device application aimed at enhancing skin ability to reconstruct its damaged sites through a proper dermal regenerative process. In particular, Q-switched Nd-YAG laser (Medlite C6 laser, Conbio, USA) applied with a fluence of 8 J/cm^2^, a pulse width of 5 ns, and a spot size of 4 mm exerts a photo-mechanical action that improve skin repair. Besides, hyaluronan hybrid cooperative complexes (HCC) widely exploited in dermoesthetic applications proved specific actions on keratinocytes and fibroblasts monolayer repair. We evaluated this specific laser treatment in vitro on a wound healing model based on human keratinocytes (HaCaT) alone and in combination with HCC. In addition, we evaluated key biomarkers of dermal repair. Scratched HaCaT monolayers were treated with laser and successively with HA-based formulations (HHA and HCC). For each treatment and the control samples, at least 3 different wells were analyzed. Wound closure was quantified, measuring five view filed for each well at increasing incubation time, exploiting time lapse videomicroscopy and image analysis, permitting to compare the different healing rate of treatments respect to control. By real-time PCR and western blotting, we evaluated biomarkers of wound regeneration, such as integrins, aquaporin three (AQP3), and proinflammatory cytokines. The ANOVA test was used to assess statistical significance of the results obtained. Laser-treated cells achieved wound closure in about 37 h, faster than the control, while when coupled to HCC, the complete reparation was obtained in 24 h. Integrin αV was upregulated by treatments, with in particular about four-fold increase respect to the control when HCC + laser was used. In addition, integrin β3 was upregulated by all treatments especially with the combination of laser and HCC proved more efficient than others (~ 14-folds). A slighter but significant increase of AQP3 gene expression of 61% was found for laser treatment while the latter combined with HCC determined an upregulation of 72%. By coupling laser treatment and HCC, further healing improvement and consistent biomarker modulation was observed. Our results may support clinical implementation of new dermatology protocols conjugating laser treatments with topical or injective HA formulations as a valid tool in treatments to repair scars or other skin defects.

**Electronic supplementary material:**

The online version of this article (10.1007/s10103-020-03145-5) contains supplementary material, which is available to authorized users.

## Introduction

Wound healing is a physiological dynamic complex response to tissue damage, which consists of three overlapping phases: inflammation, tissue formation, and tissue remodeling [1]. The mechanisms underlying each phase have been widely explored thanks to the recent advances in cellular and molecular biology. It is well established that a pivotal role is played by a superbly orchestrated cross-talk among blood cells, parenchymal cells, soluble mediators, and extracellular matrix, which lead together to proper wound repair and tissue regeneration, restoring the skin barrier [1]. Particularly each phase includes specific combinations of signals such as growth factors, cytokines, matrix metalloproteinases, and their inhibitors [2]. Aquaporins (AQPs) are intrinsic membrane proteins involved in water transport, among them aquaporin 3 (AQP3) is also involved in glycerol transport reported as “aquaglyceroporin,” localized in the skin and specifically to the basal epidermis layer [3] and sebaceous glands [4]; however, AQP3 promotes the migration and proliferation of keratinocytes during healing [3]. Most of them may act as “start signals” able to trigger relatively sedentary cell lineages at the wound margin, to fill the wound site, prompting proliferation and synthesis of new extracellular matrix [2].

During the last years, several attempts have been made to improve the wound healing process, thus making skin able to reconstruct its damaged sites better, as occurs in regeneration.

To date, the ability of thermal lasers to improve skin wound healing has been studied, for both scar revision and scar prevention immediately after surgery (Laser-Assisted Skin Healing, LASH), investigating the proper wavelength and pulse width [5]. The resurfacing capability of ablative lasers (CO_2_ and Er:YAG laser) has been used for scar revision, while diode lasers and recently Nd:YAG lasers have been employed for LASH, due to their remodeling abilities [6, 7]. Besides, to select the correct wavelength, which acts on the penetration power and on the target structures, it is necessary to calibrate the pulse duration that determines the heat source generated in the tissue, the time available for heat diffusion, and the mechanism of laser-tissue interactions, photo-thermal vs photo-mechanical effect [6, 7].

It is well worth noting that the main mechanism by which the Q-switched Nd:YAG laser exerts its action on tissues is the photo-mechanical effect, while that exert by the long-pulsed Nd:YAG laser is the photo-thermal effect [7, 8]. The neodymium-doped yttrium aluminum garnet (Nd:YAG laser) is a laser that issues various wavelengths and can operate in continuous, long-pulsed, Q-switched, or potassium titanyl phosphate (KTP) modes. Q-switched Nd:YAG laser 1064 nm is able to penetrate the tissue more deeply and reduce tissue damage. Thanks to its shorter pulse width, Q-switched Nd:YAG laser delivers, selectively in the upper dermis, higher doses of energy and stimulates cellular metabolism, with lower temperature and thus damages for epidermidis [7, 8]. A number of laser devices and light sources, emitting at various wavelengths through non-ablative mechanisms, have been described that effectively improve the appearance of aged skin. Narrow bands of visible light (400–800 nm LED) on photo-rejuvenation were specifically investigated with interesting clinical results [9]. Previous studies have been showed that the photo-mechanical effect would promote more effectively the synthesis of collagen type III, while the photo-thermal effect would elicit more sharply the formation of collagen type I [8]. Being collagen type III, the predominant isotype of fetal and youth skin gradually replaced by collagen type I during the aging process, it may play a pivotal role in skin remodeling [10].

Furthermore, Q-switched Nd:YAG laser proved efficacious in improving the organization of collagen fibrils: its photo-mechanical effect promotes the shift from disorganized collagen with reduced affinity for stain to parallel well-oriented fibers with enhanced staining. Being the orientation of the fibrous matrix, the major difference among normal tissue and scar tissue, Q-switched Nd:YAG laser would be more effective in reducing scars formation eventually erasing this healing issue [5, 10].

A literature report of studies performed with Q-switched 1064 nm Nd:YAG laser, histologic analysis of the laser-treated skin areas showed evidence of dermal remodeling along with epidermal hyperplasia, new collagen formation, an increase in the number of fibroblasts, and angiogenesis [11]. Schmults et al. (2004) showed treatment with Q-switched 1064 nm Nd:YAG laser prompted the formation of new collagen and slight fibrosis on the dermis, without damage to the epidermis by performing histological studies, after [12].

In this study, we aimed at evaluating the potential synergic effect of laser treatment with a well-known glycosaminoglycan widely exploited in skin care, namely hyaluronic acid (HA). In particular, we evaluated, besides the well-known high molecular weight hyaluronan gels, the effect of hybrid cooperative complexes based on HA at different molecular weight (HCC) (e.g., NaHyCO® Technology) combined to the laser treatment in vitro wound healing model. Briefly, HCC were developed to contemporary benefit of the biological function of low MW HA and high MW HA, also generating through a thermal treatment these complexes that permit a prolonged resistance to enzyme-mediated degradation, as previously demonstrated [13, 14]. Furthermore, hydrodynamic and rheological characteristics of HCC proved different from the simple mixture of the two components [15].

HCC was widely studied in vitro and in vivo on its potentialities in dermoesthetic [13, 15]. The recent literature reported a faster reparation of an in vitro scratched cellular monolayer, in presence of HCC respect to other HHA-based gels. Specific remodeling and inflammation biomarkers (MMPs, TGFβ, TNFα) also proved modulated, confirming the positive effect of HCC in prompting the wound closure [13].

Laser treatment activates healing by different mechanisms respect to HA topical/injective treatment, in this respect, especially regarding scar treatment or specific wound repair, the coupling of the two treatments might be promising.

In this experimental work, we set up an in vitro model (a) to compare and quantitatively evaluate the performance of laser and laser coupled to HA gels in prompting healing and (b) to unravel the mechanism beyond the remodeling process when using the single treatments (either laser exposure, or HA gels) and coupling of the two approaches. Our hypothesis was that given the beneficial role of hyaluronan treatments, and the remodeling effect recently assessed for Q-switched laser applications, protocols combining the two strategy may fasten and improve healing process. To achieve this goal, in vitro scratch model on human keratinocytes was used for time lapse videomicroscopy analyses of the repair kinetic. In addition, gene (RT-PCR) and protein (western blotting) expression of specific biomarkers were quantitatively evaluated.

## Materials and methods

### Cell culture and treatments

HaCaT cells were cultured in Dulbecco’s minimal essential medium (DMEM; Gibco-BRL, Milan, Italy) supplemented with 10% fetal calf serum (FCS; VWR, Milan, Italy), 1% glutamine, and 1% pen-strep (Lonza, Basel, Switzerland) at 37 °C in a 5% CO_2_ humidified atmosphere. In our experiment, cells were used at 70–80% confluence. HaCaT cells were irradiated or not with 1064 nm Q-switched Nd:YAG laser (Medlite C6 laser, Conbio, USA) at a fluence of 8 J/cm^2^, a pulse width of 5 ns, and a spot size of 4 mm. The optical arm was positioned at a distance of 2.5 cm from the cells and laser irradiation was carried out twice at an interval of 1 s.

#### High molecular weight hyaluronic acid sodium salt lot

N. 02622 (HHA) (1120 ± 100 kDa, Altergon, ultrapure SHYALT) and low molecular weight HA were kindly provided by Altergon srl (Morra De Sanctis, Avellino, Italy). The product was extensively characterized by Size Exclusion Chromatography-Triple Detector Array (SEC-TDA) equipment by Viscotek (Lab Service Analytica S.R.L., Rome, Italy) as reported elsewhere [14, 16, 17]. The HCC complex (containing 16 + 16 g/L) (PROFHILO) was obtained following the NAHYCO® technology procedure as previously described [10, 13, 17]. The HHA/LHA complex formation, named HCC, was ascertained by performing viscosity measurements according to the literature (data not shown) [15].

### In vitro scratched test assay in standardized condition or modified

Wound healing was evaluated through in vitro scratch assay performed on HaCaT monolayers and monitored by time lapse videomicroscopy station for 48–72 h (TLVM, Okolab, Naples, Italy).

The protocols were reported in our previous manuscript [15]. Differently, in this specific study, scratched HaCaT monolayers were treated with laser eventually followed HA gel treatment. Specifically, either H-HA or HCC, at 0.32% w/w, were added in DMEM 1% FBS. The scratched monolayer, incubated with fresh serum-supplemented medium (1% v/v FBS), was used as a control. The multiwell containing all the treated samples and the controls (at least in duplicate) was placed in the CO_2_ stage incubator and wound closure was monitored by selecting representative field of view, in a number of 5 for each well. The images of “wound closure” phenomenon, captured by a CCD camera (ORCA ER, Hamamatsu Photonics, Hamamatsu City, Japan), processed with a software (OKO-Vision 4.3, OKOLAB, Naples, Italy) by allowing us to analyze the experiment by displaying the recorded images and to perform quantitative analysis of wound healing. Duplicates performed for each scratch assay.

### Cell viability measurement

The viability of HaCaT after irradiation with laser was measured by the MTT procedure (Alpha Kit, Biochrom, Berlin, Germany), which is a colorimetric assay for cellular growth and survival using 3-(4,5-dimethylthiazol-2-ly)-2,5-diphenyltetrazoliumbromide (Roche Diagnostic, Basel, Switzerland). An MTT-dye solution was added to each well and the incubation was continued for 180 min. Following the MTT procedure, the medium was aspirated from each well and replaced with isopropanol to dissolve the formazan crystals formed in viable metabolic active keratinocytes. The content of each well was mixed for 5 min by shaking the plates. The plates were then examined spectrophotometrically [18]. The viability was calculated by measuring the increase in absorbance at 570 nm and was expressed as a percentage of the control value.

Values are the mean ± standard deviation (SD) of three replicates in three different experiments.

### Real-time PCR analysis

Confluent keratinocytes (10^6^/well) were subjected or not to wound healing evaluation after or not irradiation with laser. After 24, 48 h total RNA was isolated using the High Pure RNA Isolation Kit (Roche, Milan, Italy). Two hundred nanograms of total cellular RNA was reverse-transcribed (Expand Reverse Transcriptase, Roche, Milan, Italy) into complementary DNA (cDNA) using random hexamer primers (Random hexamers, Roche, Milan, Italy) at 42 °C for 45 min, according to the manufacturer’s instructions. Real-time PCR was carried out with the LC Fast Start DNA Master SYBR Green kit (Light Cycler 2.0 Instrument, Roche, Milan, Italy) using 2 ml of cDNA, corresponding to 10 ng of total RNA in a 20-ml final volume, 3 mM MgCl_2_, and 0.5 mM sense and antisense primers (Table 1). A melting curve was made at the end of each amplification to ensure the absence of non-specific reaction products. The accuracy of mRNA quantification depends on the linearity and efficiency of the PCR amplification. Both parameters were assessed using standard curves generated by increasing amounts of cDNA. Quantification is based on the threshold-cycle values, which are measured in the early stage of the exponential phase of the reaction, and on normalization to the internal standard curve obtained with the house keeping b-actin gene to avoid discrepancies in input RNA or in the reverse transcription efficiency. The PCR products were examined on 1.4% agarose gel.

**Table 1:** Real-time PCR carried out using sense and antisense primers

Gene	Forward and reverse primer	Thermal cycles	Cycles *N* (bp)
*AQP3*	5′-CTCCAGCATCCGACAAGAAGC-3′ 5′-GAGGTCGTAGGCTGTTCTTCG-3′	5″ at 94 °C, 12″ at 58 °C, 28″ at 72 °C for 40 cycles	280
*Integrin αV*	5′-TAAGGCAGATGGCAAAGGAG-3′ 5′-CAGTGGAATGGAAACGATGAGC-3′	5″ at 94 °C, 10″ at 64 °C, 20″ at 72 °C for 40 cycles	510
*Integrin β3*	5′-GGTGCAATGAAGGGCGTGTTGG-3′ 5′--3′	5″ at 95 °C, 14″ at 57 °C, 26″ at 72 °C for 40 cycles	440
*TNF-α*	5′-CAGAGGGAAGAGTTCCCCAG-3′ 5′-CCTTGGTCTGGTAGGAGACG-3′	5″ at 95 °C, 6″ at 57 °C, 13″ at 72 °C for 40 cycles	324
*IL-1α*	5′-CATGTCAAATTTCACTGCTTCATCC-3′ 5′-GTCTCTGAATCAGAAATCCTTCTATC-3′	5″ at 95 °C, 8″ at 55 °C, 17″ at 72 °C for 45 cycles	421
*IL-1β*	5′-GCATCCAGCTACGAATCTCC-3′ 5′-CCACATTCAGCACAGGACTC-3′	5″ at 95 °C, 14″ at 58 °C, 28″ at 72 °C for 40 cycles	708
*TGF-β*	5′-CCGACTACTACGCCAAGGAGGTCAC-3′ 5′-AGGCCGGTTCATGCCATGAATGGTG-3′	5″ at 94 °C, 9″ at 60 °C, 18″ at 72 °C for 40 cycles	439

### Western blot analysis

For the evaluation of western blotting, HaCaT cells were treated with HHA or HCC for 48 h with and without laser treatment. To estract nuclear and cytoplasmic protein fraction Ne-PER Kit was used (Nuclear and Cytoplasmic Extraction reagents, Thermofisher, Waltham, MA USA 02451) to isolate nuclear and cytoplasmic protein fractions. Protein concentrations were determined using the Bio-Rad protein assay reagent (Bio-Rad Laboratories, Milan, Italy) (Bio-Rad Protein Assay Dye Reagent Concentrate, Bio-Rad Laboratories, Marnes-la-Coquette, France). Equal amounts of proteins (20 μg) were loaded on a SDS-PAGE and transferred them to a nitrocellulose membrane (Protran™ NC Nitrocellulose Membranes, Amersham™, GE Healthcare, Milano, Italy). The filters were incubated with antibodies against integrin αV (mouse monoclonal IgG P2W7 sc-9969, Santa Cruz Biotechnology, CA, USA, 1:200 v/v), integrin β3 (integrin αV mouse monoclonal IgG P2W7 sc-9969, Santa Cruz Biotechnology, CA, USA, 1:200 v/v), AQP3 (mouse monoclonal IgG F-1 Santa Cruz Biotechnology, CA, USA 1:200 v/v), and actin (actin goat polyclonal IgG I-19 Santa Cruz Biotechnology, CA, USA 1:500) at room temperature for 2 h. Membranes were washed three times for 10 min and incubated with a 1:5000 dilution of horseradish peroxidase-conjugated anti-mouse antibodies and with a 1:10,000 dilution of horseradish peroxidase-conjugated anti-goat antibodies for 1 h, respectively.

All secondary antibodies are obtained from Bethyl Laboratories. Blots (Montgomery, TX USA) were developed using the ECL system according to the manufacturer’s protocols (Amersham Biosciences). Actin antibody was used as the gel loading control.

### Statistical analysis

Each experiment was performed at least three times except for western blotting that were repeated twice. The results are expressed as mean ± standard deviations (SD). The significant differences among the groups were assessed using one-way ANOVA and Tukey post hoc test for comparing a family of six estimates by JASP software (JASP 0.13.0.0, Amsterdam, The Netherlands).

Significative differences were appointed and marked on figure (e.g., *, #, §, etc.) for *p* values lower than 0.05, for the different compared groups and tables containing the complete results are reported as supporting information.

## Results

### Effect on cell viability

**Fig. 1:**
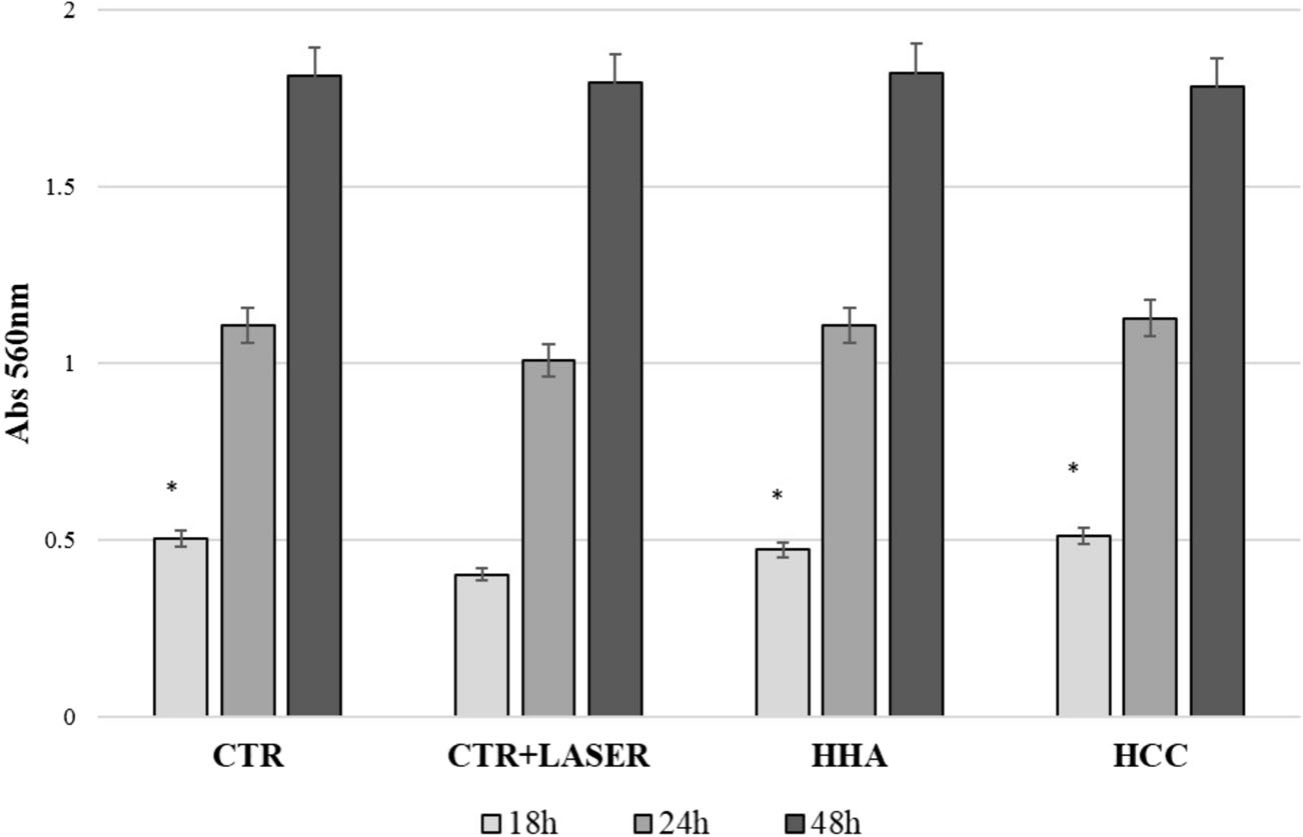
Cell viability measurement. HaCaT viability after irradiation with laser and after treatment with HCC and H-HA measured by the MTT assay. Results were considered significantly different for *p*tukey < 0.05 # vs CTR, * vs CTR + LASER, § vs HCC, ° vs HCC + LASER, $ vs HHA, & vs HHA + LASER for each of the 4 families compared (tables in supplementary files)

Q-switched Nd:YAG laser used at 1064 nm at 8 J/cm^2^ and respectively HCC and H-HA did not significantly influence HaCaT morphology and viability (Fig. 1) as demonstrated by optical microscopy observation and MTT assay after 18, 24, and 48 h after irradiation/HA gel addition. At 18 h incubation, ANOVA test highlighted a significative difference in viability of hyaluronan-treated cells respect to laser; however, at 48 h, ANOVA test applied for a family of 4 proved there were no significative differences among groups.

### Q-switched 1064 nm Nd:YAG laser enhances effect of hyaluronans on wound healing

**Fig. 2:**
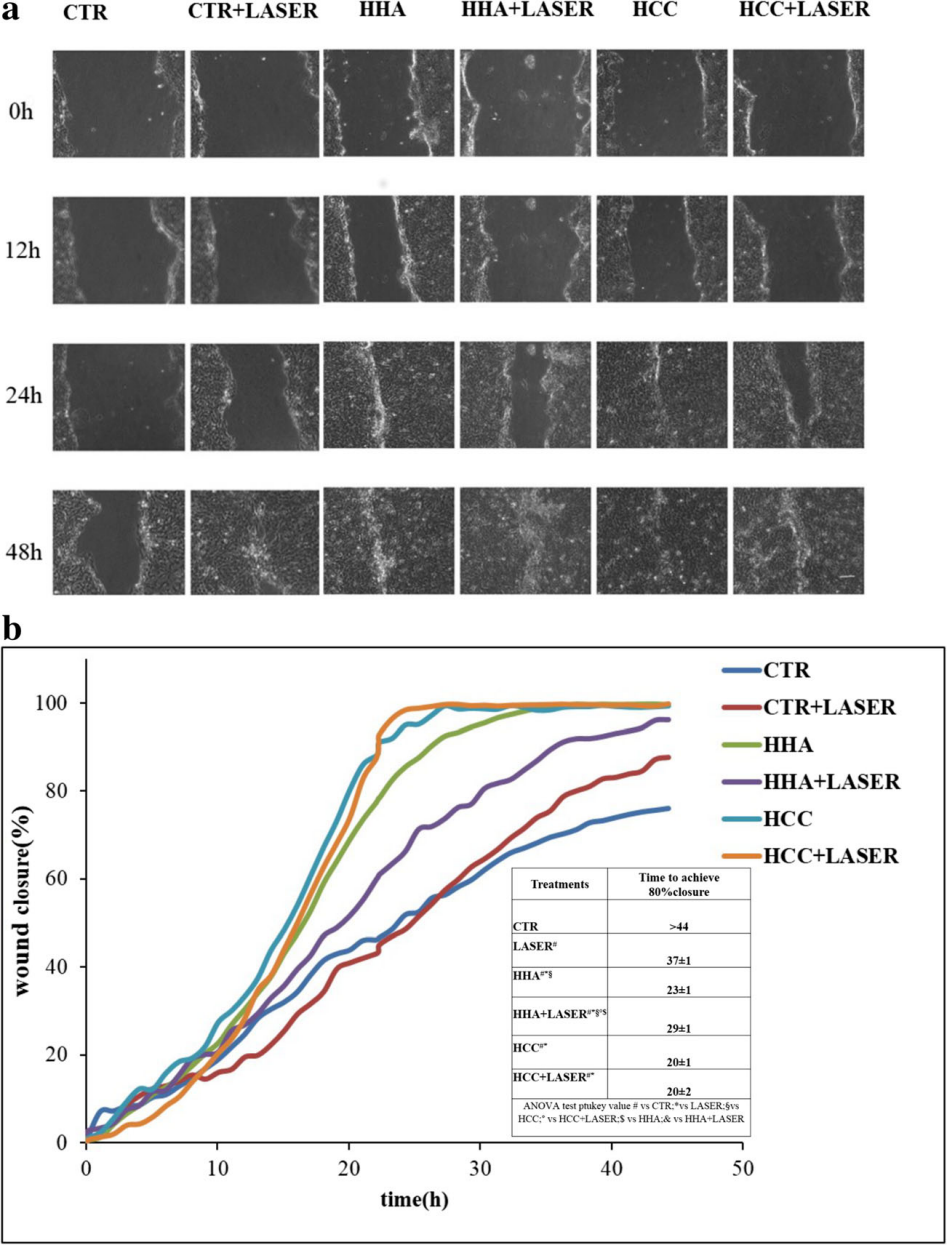
Wound healing assay. **a** Representative images of HaCaT wound healing in the CTR and in presence of treatments. **b** Reparation area percentage [Areat_0_ − Area_t_/Areat_0_ × 100] for the control and in presence of the treatments. The curves are averages of three different experiments with standard deviation within 5% of the value. Results were considered significantly different for *p*tukey < 0.05 # vs CTR, * vs CTR + LASER, § vs HCC, ° vs HCC + LASER, $ vs HHA, & vs HHA + LASER for each of the 5 families compared (tables in supplementary files)

Concerning in vitro scratch test, Fig. 2a shows the panel of representative images of the healing process in the control and in the treated samples. The wound closure curves (average at least of 5 view fields of each well) relative to the control and treated samples were reported in Fig. 2b. HA-treated samples, in particular for HCC, prompted wound closure faster coherently to results previously described [15]. We observed differences between CTR and laser-treated cells, after 24 h incubation, while after 48 h cell, the control wells still presented a residual scratch. In particular, laser treatment fastened the reparation closing 80% of the scratch at about 37 ± 1 h. It could be argued that the beneficial effect of laser treatment has a delay time, initially resulting in a slower migration phenomenon (i.e., 12 h). It is evident that the scratch closure occurred at a significant faster rate in the presence of HCC and laser + HCC without appreciable difference between the two; wound closure was reached approximately in 24 h. HCC-treated samples reached the 80% of the scratched area repair in half the time respect to the control corroborating the immediate efficacy of HCC in the in vitro model tested.

### Effect of Q-switched Nd:YAG laser and hyaluronans on cytokine modulation of HaCaT cells during the healing process

**Fig. 3:**
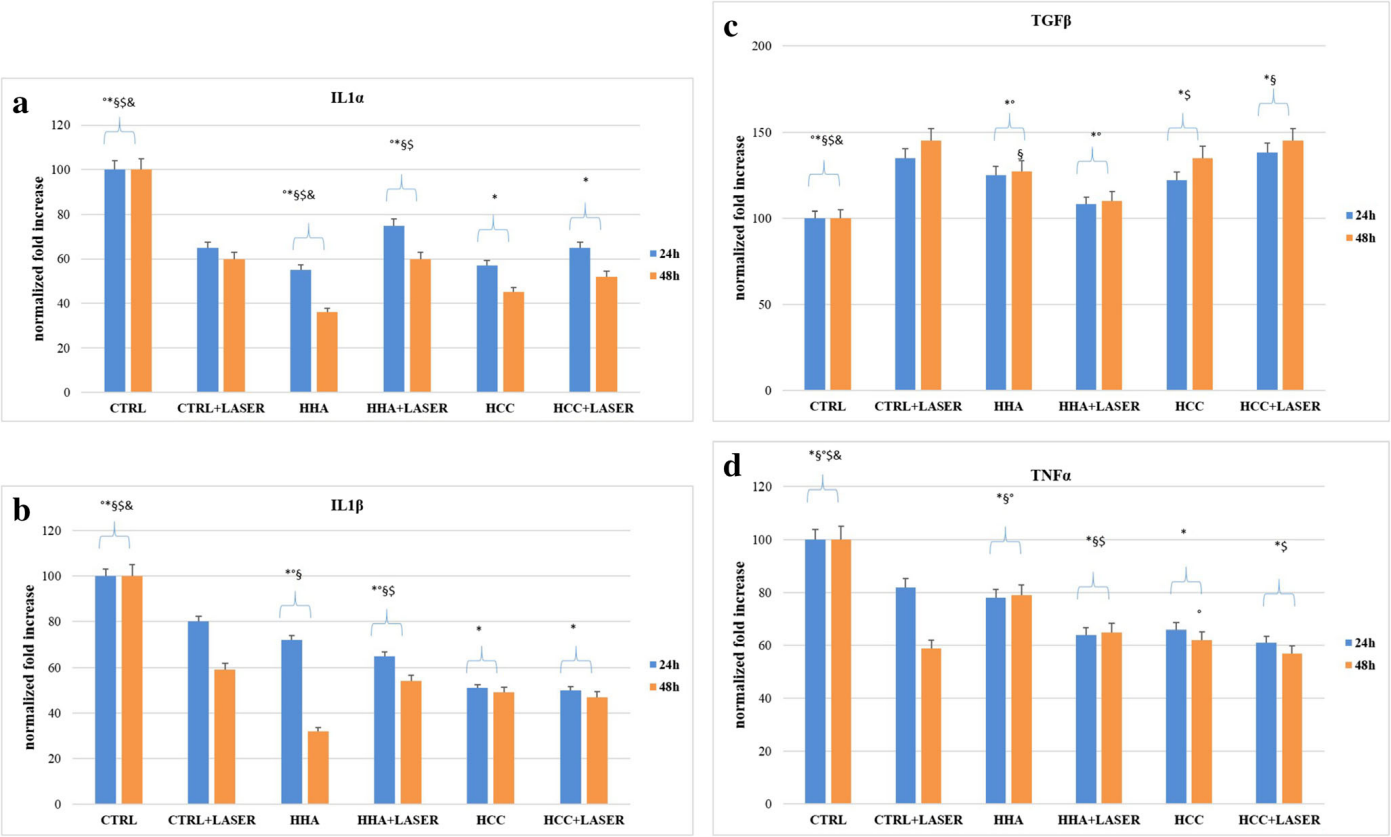
Real-time PCR analysis using specific primers for cytokines. **a** Relative IL-1α gene expression from HaCaT irradiated with laser and after treatment with HCC and H-HA. **b** Relative IL1β, gene expression from HaCaT irradiated with laser and after treatment with HCC and H-HA. **c** Relative TNFα gene expression from HaCaT irradiated with laser and after treatment with HCC and H-HA. **d** Relative TGFβ gene expression from HaCaT irradiated with laser and after treatment with HCC and H-HA. Results were considered significantly different for *p*tukey < 0.05 # vs CTR, * vs CTR + LASER, § vs HCC, ° vs HCC + LASER, $ vs HHA, & vs HHA + LASER for each of the 6 families compared (tables in supplementary files)

To evaluate the ability of laser to modulate the proinflammatory response in HaCaT cells after wound, the gene expression of the proinflammatory cytokines IL-1 alpha, IL-1 beta, and TNF-alpha was analyzed. HaCaT cells did not express basal level of these cytokines. As shown in Fig. 3a–c, HaCaT cells after 24 and 48 h from the scratches displayed high levels of cytokine gene expression.

A major reduction was observed after 48 h from treatment with laser, in particular gene expression of IL-1 alpha, IL-1 beta, and TNF-alpha were downregulated respectively of 40% and of 42% in respect to control. The combined use of HCC and laser determined a reduction of IL-1 alpha of 48%, IL-1 beta of 52%, and TNF-alpha of 45%. The treatment with laser and laser + HCC showed an upregulation of TGF-β1 after 24 h, with an evident increase at 48 h, too (Fig. 3d). When ANOVA test for a family of 6 released a *p* value lower than 0.05, significative differences were marked in the figure with different symbols (e.g., *, #, §, etc.) as described in the legend.

### HHA and HCC upregulate AQP3 in irradiated and scratched HaCaT cells

**Fig. 4:**
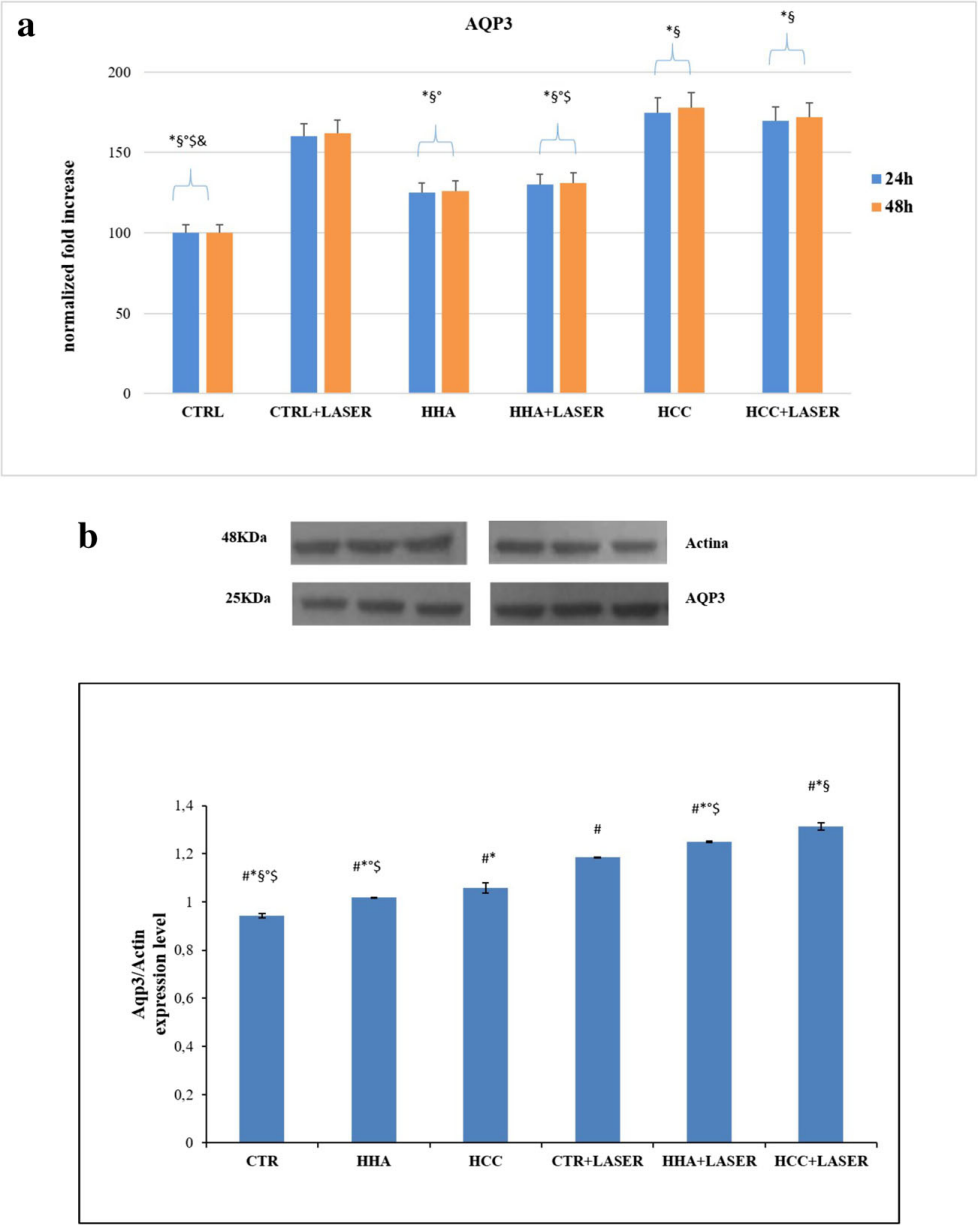
Real-time PCR and western blot analysis. **a** Relative AQP3 gene expression from HaCaT irradiated with laser and after treatment with HCC and HHA. *p < 0.01 for CTR + LASER, HCC, HCC + LASER vs CTR; #*p* < 0.05 for HHA and HHA + LASER vs CTR; #*p* < 0.05 for HCC and HCC + LASER vs CTR + LASER; a slight but significant difference was also found for HCC + LASER vs HHA + LASER. **b** AQP3 protein expression level and densitometric results were normalized in respect to actin. All values were expressed in the form of mean ± SD (*n* = 3). Results were considered significantly different for *p*tukey < 0.05 # vs CTR, * vs CTR + LASER, § vs HCC, ° vs HCC + LASER, $ vs HHA, & vs HHA + LASER for each of the 6 families compared (tables in supplementary files)

To determine the potential effect of laser treatment laser on skin barrier, we examined gene expression of AQP3 at 24, 48 h after irradiation. In Fig. 4a it is shown that both after 24 and 48 h laser induced an increase of AQP3 expression of 61%, while laser combined with HCC determined an upregulation of 72%. These data were confirmed by western blot. There was an increase for all treatments, in particular in presence of laser combination. HCC + laser was the best treatment to prompt AQP3 increment of about 1.3-fold increase respect to the control (for control, we indicate HaCaT cells without treatment and irradiation), thus suggesting a beneficial effect on skin hydration (Fig. 4b).

The significant differences are highlighted in figures and ANOVA test output can be found additionally in supporting information files.

### Hyaluronans and laser upregulate αV and β3 integrins in scratched HaCaT cells

**Fig. 5:**
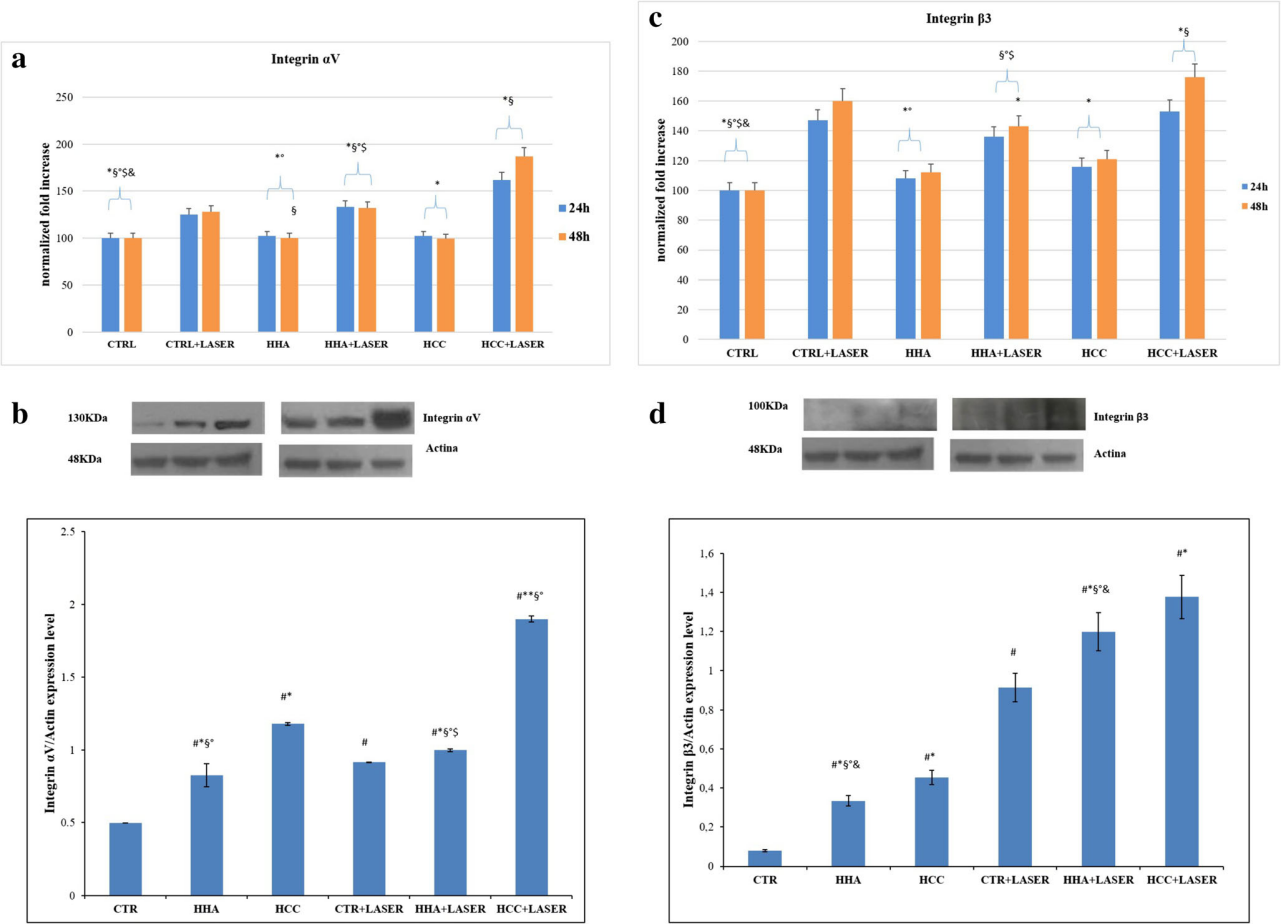
Real-time PCR analysis and western blot analysis. **a** Relative integrin αV gene expression from HaCaT irradiated with laser and after treatment with HCC and HHA. **b** Integrin αV protein expression level and densitometric results were normalized in respect to actin. All values were expressed in the form of mean ± SD (*n* = 3). **c** Relative integrin β3 gene expression from HaCaT irradiated with laser and after treatment with HCC and H-HA. **d** Integrin β3 protein expression level and densitometric results were normalized in respect to actin. All values were expressed in the form of mean ± SD (*n* = 3). Results were considered significantly different for *p*tukey < 0.05 # vs CTR, * vs CTR + LASER, § vs HCC, ° vs HCC + LASER, $ vs HHA, & vs HHA + LASER for each of the 6 families compared (tables in supplementary files)

To investigate the role of adhesion molecules on re-epithelization, we analyzed gene expression of integrin αV and β3, as reported in Fig. 5a and c. Integrin αV and β3 gene expression was upregulated with an evident increase after combined treatment (laser + HCC). These results were confirmed by western blot analysis. Integrin αV was upregulated in particular of about 3.8-folds by HCC + laser respect to the control (Fig. 5b). Integrin β3 is increased by all treatments especially in presence of laser. The combination laser + HCC proved more efficient than others, showing about 14-fold increase respect to the control (Fig. 5d). The significant differences are highlighted in figures and ANOVA test output can be found additionally in supporting information files.

## Discussion

Scratch assay is a consolidated practice in our laboratory and TLVM helps not only to empirically follow biological phenomena but also to quantify some of those. In this way, in a single multiwell, different events may be analyzed and diverse treatments compared contemporary on the same human cells (same passage, same incubation conditions). For the first time, Q-switched 1064 nm Nd:YAG laser treatments were compared to control in scratched HaCaT monolayer. Preliminary viability evaluation with MTT test showed that there is an initial stress caused by laser treatment that in fact reduced metabolic activity of cells treated respect to the ones added with hyaluronan or control. This finding is in agreement with the slower reparation rate found in wound healing assay, at least in the first 18–24 h.

Thus in this experimental model, laser treatment proved to fasten reparation. However, when we included HA-based treatments, these were the most powerful in increasing the repair rate, in fact, overall the wound closure occurred earlier. In agreement with previous studies, we confirmed that HCC is the best performing gel in keratinocytes monolayer regeneration. However, restoring the epidermis thickness and barrier integrity in wound healing require a strong integration of signaling molecules such as cytokines, growth factors, integrin, ECM, and metalloproteases. To better highlight the possibly occurring synergy between laser treatments and HA in skin repair, biochemical key biomarkers were investigated. Scratched keratinocytes, treated with laser, showed IL-1 alpha, IL-1 beta, and TNF-alpha downregulated gene expression mostly after 48 h compared to untreated controls. All HA-treated samples also reduced the cytokines tested. Finally, coupled treatments succeeded in further reduction, especially laser + HCC treatments, that for IL-1 alpha and IL-1 beta at 48 h showed the lowest expression level. TNF-alpha was reduced already at 24 h both for laser and laser + HCC treatments, specifically the latter showed a maximal 1.8-fold reduction respect to control, and an improvement of 30% respect to the sole laser treatment (Fig. 3). Proinflammatory cytokines favor the inflammatory phase of wound healing, prompting the clearance of microorganisms and stimulating the expression of growth factors. Among those, we chose to evaluate the effect of laser on TGF-beta expression, which represents the most important ligand, in keratinocyte migration, during re-epithelialization [19]. TGF-beta as anti-inflammatory cytokine reduces the persistent inflammatory state, associated at chronic wound, that alters the normal wound healing. It is a growth and differentiation factor; it acts in remodeling by stimulating the synthesis of collagen and fibronectin [20] and thus the deposition of the extracellular matrix, important for re-epithelization. In laser-treated keratinocytes, there is an increase in the gene expression of TGF beta after 24 h; HHA-treated samples after laser treatment do not show a higher expression, while laser + HCC proved slightly superior both at 24 and 48 h (Fig. 3). At these times, we found activation of αV and β3 integrins, whose expression increased until 48 h, especially for HCC in combination with laser treatment. These data were confirmed at protein level. Cytokines promote the expression of many classes of adhesion molecules. These adhesion molecules are determining factor for the diapedesis of neutrophils, including selectins and integrins which interact with those already present on the membrane surface of endothelial cells. The expression of αV and β3 during re-epithelization favors binding to fibronectin, vitronectin, and collagen to initiate epithelial migration on a provisional matrix. Integrins play a central role in cell adhesion contacts function as signaling centers, and the linkages between ECM and actin cytoskeleton allow adhesion sites to serve as traction sites for cell movement [21]. Another interesting aspect of this study concerns the modulation of AQP3 expression in scratched keratinocytes monolayers treated with laser. Besides, this specific biomarker was often investigated in the characterization of biological response of HA-based fillers [22]. The skin of deficient mice in AQP3 shows reduced hydration, reduced elasticity, and delayed barrier formation as a result of damage [23]. In our previous study about effects of laser on markers of rejuvenation [24], we have demonstrated for the first time that laser increases AQP3 gene expression in human keratinocytes, favoring moisturizing and barrier function of skin. Among its different functions, AQP3 promotes the migration and proliferation of keratinocytes during healing [25, 26]. In fact, wound closure was delayed in AQP3-deficient keratinocytes. Hara and collaborators have, furthermore, showed that glycerol administration in the diet corrects healing delay in AQP3-null mice, favoring not only migration but also proliferation of keratinocytes [26]. We investigated AQP3 expression that was significantly increased by laser treatments compared to untreated controls; further increment was found by coupling HCC treatments to laser. The increase proved time dependent and the maximum expression was observed at 48 h. AQP3 upregulation suggests that laser effects are expressed not only on accelerated healing of the wound, but also on hydration, elasticity, and restoration of the barrier during skin care, as supported also by protein expression level. Q-switched laser on scratched HaCat and fibroblast monolayers was used in previous studies [27]. However, this is the first time that time lapse videomicroscopy is used to quantify healing rate, that laser treatment is combined to HA formulation, and that a whole array of biomarkers is assessed in these experimental conditions both with RT-PCR and western blotting. In our opinion, the most interesting part of this research resides into the potential synergic effect of the laser treatments with HCC. The latter are used in dermatology and esthetic medicine; however, being a recently proposed formulation and having showed peculiar and interesting behavior on fibroblast and stem cells are good candidates to new approaches and combined clinical treatments.

In fact, our results suggest that these treatments may contribute to enhance tissue repair, to reduce chronic inflammation in the wound, as previously reported [27, 28]. Besides, laser treatment was already reported to favor hydration and to enhance antimicrobial molecules expression (HBD-2), also HCC proved efficient in this respect [27, 28], and in prompting migration [29]. Due to these results and the supportive previous scientific literature, complications due to cells overgrowth as hypertrophic or keloid scarring can be solved with supportive therapies, such as the use of Q-switched Nd:YAG laser eventually coupled to HA and above all HCC treatments. Excessive scarring can dramatically influence the patient’s quality of life both physically and psychologically.

These results may support clinical dermatologist to assess new protocols conjugating laser treatments with the application of topical or injective HA-based gels to improve bioremodelling in scars/skin defects/keloids/fibrotic scars. Resolving the excessive inflammation, the laser treatments activate stop signals that block further formation of fibrosis or hypertrophic scar.

## Conclusions

The use of Q-switch laser in combination with different formulation of hyaluronic acid represents an important dermatological research area for the identification of effective innovative combination therapies, both in esthetic and regenerative medicines. The results showed improved repair rate in presence of HCC beyond all treatments and the positive modulation of all the key biomarkers analyzed. The present study may be supportive in translational medicine and specific clinical protocols may be assessed for targeted clinical trials.

## Electronic supplementary material

ESM 1 (DOCX 93 kb)
