# Supplementary material for: Q-switched Nd-YAG laser alone and in combination with innovative hyaluronic acid gels improve keratinocytes wound healing in vitro
Source: Lasers Med Sci. 2020 Sep 26;36(5):1047–57. doi: 10.1007/s10103-020-03145-5 (PMC8222021; doi:10.1007/s10103-020-03145-5)
Supplement: Supplementary file 1 — (DOCX 93 kb) [file 10103_2020_3145_MOESM1_ESM.docx]

# vs CTR

*vs CTR+LASER or LASER

§vs HCC

° vs HCC+LASER

$ vs HHA

& vs HHA+LASER

| **Post Hoc Comparisons - V1** | | | | | | | | | | | |
| --- | --- | --- | --- | --- | --- | --- | --- | --- | --- | --- | --- |
|  | |  | | **Mean Difference** | | **SE** | | **t** | | **p _tukey_** | |
| LASER |  | HCC |  | -0.111 |  | 0.017 |  | -6.373 |  | < .001 |  |
| * |  | HHA |  | -0.070 |  | 0.017 |  | -4.055 |  | 0.015 |  |
|  |  | ctr |  | -0.101 |  | 0.017 |  | -5.793 |  | 0.002 |  |
| HCC |  | HHA |  | 0.040 |  | 0.017 |  | 2.317 |  | 0.173 |  |
| § |  | ctr |  | 0.010 |  | 0.017 |  | 0.579 |  | 0.936 |  |
| HHA  $ |  | ctr |  | -0.030 |  | 0.017 |  | -1.738 |  | 0.366 |  |
|  | | | | | | | | | | | |
| *Note.*  P-value adjusted for comparing a family of 4 | | | | | | | | | | | |

**Figure 1: MTT at 18h**

| **Post Hoc Comparisons - V1** | | | | | | | | | | | |
| --- | --- | --- | --- | --- | --- | --- | --- | --- | --- | --- | --- |
|  | |  | | **Mean Difference** | | **SE** | | **t** | | **p _tukey_** | |
| LASER |  | HCC |  | -0.121 |  | 0.040 |  | -3.035 |  | 0.063 |  |
| * |  | HHA |  | -0.101 |  | 0.040 |  | -2.529 |  | 0.129 |  |
|  |  | ctr |  | -0.101 |  | 0.040 |  | -2.529 |  | 0.129 |  |
| HCC |  | HHA |  | 0.020 |  | 0.040 |  | 0.506 |  | 0.955 |  |
| § |  | ctr |  | 0.020 |  | 0.040 |  | 0.506 |  | 0.955 |  |
| HHA  $ |  | ctr |  | -4.649e -16 |  | 0.040 |  | -1.168e -14 |  | 1.000 |  |
|  | | | | | | | | | | | |
| *Note.*  P-value adjusted for comparing a family of 4 | | | | | | | | | | | |

**Figure 1 : MTT at 24h**

| **Post Hoc Comparisons - V1** | | | | | | | | | | | | | | | | |  |
| --- | --- | --- | --- | --- | --- | --- | --- | --- | --- | --- | --- | --- | --- | --- | --- | --- | --- |
|  | |  | | | **Mean Difference** | | | **SE** | | | **t** | | | **p _tukey_** | | |  |
| LASER |  | | HCC |  | | 0.010 |  | | 0.066 |  | | 0.153 |  | | 0.999 |  | |
| * |  | | HHA |  | | -0.030 |  | | 0.066 |  | | -0.458 |  | | 0.966 |  | |
|  |  | | ctr |  | | -0.020 |  | | 0.066 |  | | -0.305 |  | | 0.989 |  | |
| HCC |  | | HHA |  | | -0.040 |  | | 0.066 |  | | -0.611 |  | | 0.926 |  | |
| § |  | | ctr |  | | -0.030 |  | | 0.066 |  | | -0.458 |  | | 0.966 |  | |
| HHA  $ |  | | ctr |  | | 0.010 |  | | 0.066 |  | | 0.153 |  | | 0.999 |  | |
|  | | | | | | | | | | | | | | | | |  |
| *Note.*  P-value adjusted for comparing a family of 4 | | | | | | | | | | | | | | | | |  |

**Figure 1 : MTT at 48h**

| **Post Hoc Comparisons - V1** | | | | | | | | | | | |
| --- | --- | --- | --- | --- | --- | --- | --- | --- | --- | --- | --- |
|  | |  | | **Mean Difference** | | **SE** | | **t** | | **p _tukey_** | |
| CTR+LASER |  | HCC |  | 17.067 |  | 1.152 |  | 14.820 |  | < .001 |  |
| * |  | HCC+LASER |  | 16.733 |  | 1.152 |  | 14.531 |  | < .001 |  |
|  |  | HHA |  | 14.033 |  | 1.152 |  | 12.186 |  | < .001 |  |
|  |  | HHA+LASER |  | 7.800 |  | 1.152 |  | 6.773 |  | < .001 |  |
| HCC |  | HCC+LASER |  | -0.333 |  | 1.152 |  | -0.289 |  | 0.998 |  |
| § |  | HHA |  | -3.033 |  | 1.152 |  | -2.634 |  | 0.137 |  |
|  |  | HHA+LASER |  | -9.267 |  | 1.152 |  | -8.047 |  | < .001 |  |
| HCC+LASER |  | HHA |  | -2.700 |  | 1.152 |  | -2.345 |  | 0.208 |  |
| ° |  | HHA+LASER |  | -8.933 |  | 1.152 |  | -7.758 |  | < .001 |  |
| HHA  $ |  | HHA+LASER |  | -6.233 |  | 1.152 |  | -5.413 |  | 0.002 |  |
|  | | | | | | | | | | | |
| *Note.*  P-value adjusted for comparing a family of 5 | | | | | | | | | | | |

**Table of Figure 2: Time to achieve 80% closure**

| **Post Hoc Comparisons - V1** | | | | | | | | | | |  |
| --- | --- | --- | --- | --- | --- | --- | --- | --- | --- | --- | --- |
|  | |  | | **Mean Difference** | | **SE** | | **t** | | **p _tukey_** |  |
| CTR+LASER |  | HCC |  | 8.067 |  | 1.475 |  | 5.470 |  | 0.002 |  |
| * |  | HCC+LASER |  | 8.882e -15 |  | 1.475 |  | 6.023e -15 |  | 1.000 |  |
|  |  | HHA |  | 9.867 |  | 1.475 |  | 6.691 |  | < .001 |  |
|  |  | HHA+LASER |  | -10.067 |  | 1.475 |  | -6.826 |  | < .001 |  |
|  |  | ctr |  | -34.800 |  | 1.475 |  | -23.598 |  | < .001 |  |
| HCC |  | HCC+LASER |  | -8.067 |  | 1.475 |  | -5.470 |  | 0.002 |  |
| § |  | HHA |  | 1.800 |  | 1.475 |  | 1.221 |  | 0.819 |  |
|  |  | HHA+LASER |  | -18.133 |  | 1.475 |  | -12.296 |  | < .001 |  |
|  |  | ctr |  | -42.867 |  | 1.475 |  | -29.068 |  | < .001 |  |
| HCC+LASER |  | HHA |  | 9.867 |  | 1.475 |  | 6.691 |  | < .001 |  |
| ° |  | HHA+LASER |  | -10.067 |  | 1.475 |  | -6.826 |  | < .001 |  |
|  |  | ctr |  | -34.800 |  | 1.475 |  | -23.598 |  | < .001 |  |
| HHA |  | HHA+LASER |  | -19.933 |  | 1.475 |  | -13.517 |  | < .001 |  |
| $ |  | ctr |  | -44.667 |  | 1.475 |  | -30.288 |  | < .001 |  |
| HHA+LASER |  | ctr |  | -24.733 |  | 1.475 |  | -16.771 |  | < .001 |  |
|  | | | | | | | | | | |  |
| *Note.*  P-value adjusted for comparing a family of 6 | | | | | | | | | | |  |

**Figure 3A: Gene expression of IL1-α at 24h**

| **Post Hoc Comparisons - V1** | | | | | | | | | | | |  |  |
| --- | --- | --- | --- | --- | --- | --- | --- | --- | --- | --- | --- | --- | --- |
|  |  | | **Mean Difference** | | **SE** | | **t** | | **p _tukey_** | | |  |  |
| CTR+LASER |  | HCC |  | 15.000 |  | 1.467 |  | 10.224 | |  | < .001 | |  |
| * |  | HCC+LASER |  | 7.967 |  | 1.467 |  | 5.430 | |  | 0.002 | |  |
|  |  | HHA |  | 23.967 |  | 1.467 |  | 16.335 | |  | < .001 | |  |
|  |  | HHA+LASER |  | 1.110e -15 |  | 1.467 |  | 7.567e -16 | |  | 1.000 | |  |
|  |  | ctr |  | -40.200 |  | 1.467 |  | -27.400 | |  | < .001 | |  |
| HCC |  | HCC+LASER |  | -7.033 |  | 1.467 |  | -4.794 | |  | 0.005 | |  |
| § |  | HHA |  | 8.967 |  | 1.467 |  | 6.112 | |  | < .001 | |  |
|  |  | HHA+LASER |  | -15.000 |  | 1.467 |  | -10.224 | |  | < .001 | |  |
|  |  | ctr |  | -55.200 |  | 1.467 |  | -37.623 | |  | < .001 | |  |
| HCC+LASER |  | HHA |  | 16.000 |  | 1.467 |  | 10.905 | |  | < .001 | |  |
| ° |  | HHA+LASER |  | -7.967 |  | 1.467 |  | -5.430 | |  | 0.002 | |  |
|  |  | ctr |  | -48.167 |  | 1.467 |  | -32.830 | |  | < .001 | |  |
| HHA |  | HHA+LASER |  | -23.967 |  | 1.467 |  | -16.335 | |  | < .001 | |  |
| $ |  | ctr |  | -64.167 |  | 1.467 |  | -43.735 | |  | < .001 | |  |
| HHA+LASER  & |  | ctr |  | -40.200 |  | 1.467 |  | -27.400 | |  | < .001 | |  |
|  | | | | | | | | | | | |  |  |
| *Note.*  P-value adjusted for comparing a family of 6 | | | | | | | | | | | |  |  |

**Figure 3A: Gene expression of IL1-α at 48h**

| **Post Hoc Comparisons - V1** | | | | | | | | | | |  |
| --- | --- | --- | --- | --- | --- | --- | --- | --- | --- | --- | --- |
|  | |  | | **Mean Difference** | | **SE** | | **t** | | **p _tukey_** |  |
| CTR+LASER |  | HCC |  | 29.900 |  | 1.681 |  | 17.783 |  | < .001 |  |
| * |  | HCC+LASER |  | 30.667 |  | 1.681 |  | 18.239 |  | < .001 |  |
|  |  | HHA |  | 8.433 |  | 1.681 |  | 5.016 |  | 0.003 |  |
|  |  | HHA+LASER |  | 15.833 |  | 1.681 |  | 9.417 |  | < .001 |  |
|  |  | ctr |  | -19.100 |  | 1.681 |  | -11.360 |  | < .001 |  |
| HCC |  | HCC+LASER |  | 0.767 |  | 1.681 |  | 0.456 |  | 0.997 |  |
| § |  | HHA |  | -21.467 |  | 1.681 |  | -12.767 |  | < .001 |  |
|  |  | HHA+LASER |  | -14.067 |  | 1.681 |  | -8.366 |  | < .001 |  |
|  |  | ctr |  | -49.000 |  | 1.681 |  | -29.143 |  | < .001 |  |
| HCC+LASER |  | HHA |  | -22.233 |  | 1.681 |  | -13.223 |  | < .001 |  |
| ° |  | HHA+LASER |  | -14.833 |  | 1.681 |  | -8.822 |  | < .001 |  |
|  |  | ctr |  | -49.767 |  | 1.681 |  | -29.599 |  | < .001 |  |
| HHA |  | HHA+LASER |  | 7.400 |  | 1.681 |  | 4.401 |  | 0.009 |  |
| $ |  | ctr |  | -27.533 |  | 1.681 |  | -16.375 |  | < .001 |  |
| HHA+LASER  & |  | ctr |  | -34.933 |  | 1.681 |  | -20.777 |  | < .001 |  |
|  | | | | | | | | | | |  |
| *Note.*  P-value adjusted for comparing a family of 6 | | | | | | | | | | |  |

**Figure 3B: Gene expression of IL-1β at 24 h**

| **Post Hoc Comparisons - V1** | | | | | | | | | | | |
| --- | --- | --- | --- | --- | --- | --- | --- | --- | --- | --- | --- |
|  | |  | | **Mean Difference** | | **SE** | | **t** | | **p _tukey_** | |
| CTR+LASER |  | HCC |  | 10.500 |  | 1.422 |  | 7.386 |  | < .001 |  |
| * |  | HCC+LASER |  | 12.067 |  | 1.422 |  | 8.488 |  | < .001 |  |
|  |  | HHA |  | 27.500 |  | 1.422 |  | 19.344 |  | < .001 |  |
|  |  | HHA+LASER |  | 5.433 |  | 1.422 |  | 3.822 |  | 0.023 |  |
|  |  | ctr |  | -40.867 |  | 1.422 |  | -28.746 |  | < .001 |  |
| HCC |  | HCC+LASER |  | 1.567 |  | 1.422 |  | 1.102 |  | 0.871 |  |
| § |  | HHA |  | 17.000 |  | 1.422 |  | 11.958 |  | < .001 |  |
|  |  | HHA+LASER |  | -5.067 |  | 1.422 |  | -3.564 |  | 0.035 |  |
|  |  | ctr |  | -51.367 |  | 1.422 |  | -36.132 |  | < .001 |  |
| HCC+LASER |  | HHA |  | 15.433 |  | 1.422 |  | 10.856 |  | < .001 |  |
| ° |  | HHA+LASER |  | -6.633 |  | 1.422 |  | -4.666 |  | 0.006 |  |
|  |  | ctr |  | -52.933 |  | 1.422 |  | -37.234 |  | < .001 |  |
| HHA |  | HHA+LASER |  | -22.067 |  | 1.422 |  | -15.522 |  | < .001 |  |
| $ |  | ctr |  | -68.367 |  | 1.422 |  | -48.089 |  | < .001 |  |
| HHA+LASER  & |  | ctr |  | -46.300 |  | 1.422 |  | -32.568 |  | < .001 |  |
|  | | | | | | | | | | | |
| *Note.*  P-value adjusted for comparing a family of 6 | | | | | | | | | | | |

**Figure 3B: Gene expression of IL-1β at 48 h**

| **Post Hoc Comparisons - V1** | | | | | | | | | | | |  |  |
| --- | --- | --- | --- | --- | --- | --- | --- | --- | --- | --- | --- | --- | --- |
|  |  | | **Mean Difference** | | **SE** | | **t** | | **p _tukey_** | | |  |  |
| CTR+LASER |  | HCC |  | 15.567 |  | 0.944 |  | 16.483 | |  | < .001 | |  |
| * |  | HCC+LASER |  | 20.667 |  | 0.944 |  | 21.884 | |  | < .001 | |  |
|  |  | HHA |  | 4.000 |  | 0.944 |  | 4.236 | |  | 0.011 | |  |
|  |  | HHA+LASER |  | 17.667 |  | 0.944 |  | 18.707 | |  | < .001 | |  |
|  |  | ctr |  | -18.633 |  | 0.944 |  | -19.731 | |  | < .001 | |  |
| HCC |  | HCC+LASER |  | 5.100 |  | 0.944 |  | 5.400 | |  | 0.002 | |  |
| § |  | HHA |  | -11.567 |  | 0.944 |  | -12.248 | |  | < .001 | |  |
|  |  | HHA+LASER |  | 2.100 |  | 0.944 |  | 2.224 | |  | 0.295 | |  |
|  |  | ctr |  | -34.200 |  | 0.944 |  | -36.214 | |  | < .001 | |  |
| HCC+LASER |  | HHA |  | -16.667 |  | 0.944 |  | -17.648 | |  | < .001 | |  |
| ° |  | HHA+LASER |  | -3.000 |  | 0.944 |  | -3.177 | |  | 0.068 | |  |
|  |  | ctr |  | -39.300 |  | 0.944 |  | -41.615 | |  | < .001 | |  |
| HHA |  | HHA+LASER |  | 13.667 |  | 0.944 |  | 14.472 | |  | < .001 | |  |
| $ |  | ctr |  | -22.633 |  | 0.944 |  | -23.966 | |  | < .001 | |  |
| HHA+LASER  & |  | ctr |  | -36.300 |  | 0.944 |  | -38.438 | |  | < .001 | |  |
|  | | | | | | | | | | | |  |  |
| *Note.*  P-value adjusted for comparing a family of 6 | | | | | | | | | | | |  |  |

**Figure 3C: Gene expression of TNF-α at 24 h**

| **Post Hoc Comparisons - V1** | | | | | | | | | | | |
| --- | --- | --- | --- | --- | --- | --- | --- | --- | --- | --- | --- |
|  | |  | | **Mean Difference** | | **SE** | | **t** | | **p _tukey_** | |
| CTR+LASER |  | HCC |  | -3.100 |  | 1.119 |  | -2.771 |  | 0.131 |  |
| * |  | HCC+LASER |  | 1.567 |  | 1.119 |  | 1.400 |  | 0.726 |  |
|  |  | HHA |  | -20.267 |  | 1.119 |  | -18.116 |  | < .001 |  |
|  |  | HHA+LASER |  | -6.133 |  | 1.119 |  | -5.483 |  | 0.002 |  |
|  |  | ctr |  | -41.467 |  | 1.119 |  | -37.067 |  | < .001 |  |
| HCC |  | HCC+LASER |  | 4.667 |  | 1.119 |  | 4.172 |  | 0.013 |  |
| § |  | HHA |  | -17.167 |  | 1.119 |  | -15.345 |  | < .001 |  |
|  |  | HHA+LASER |  | -3.033 |  | 1.119 |  | -2.711 |  | 0.144 |  |
|  |  | ctr |  | -38.367 |  | 1.119 |  | -34.296 |  | < .001 |  |
| HCC+LASER |  | HHA |  | -21.833 |  | 1.119 |  | -19.517 |  | < .001 |  |
| ° |  | HHA+LASER |  | -7.700 |  | 1.119 |  | -6.883 |  | < .001 |  |
|  |  | ctr |  | -43.033 |  | 1.119 |  | -38.467 |  | < .001 |  |
| HHA |  | HHA+LASER |  | 14.133 |  | 1.119 |  | 12.634 |  | < .001 |  |
| $ |  | ctr |  | -21.200 |  | 1.119 |  | -18.951 |  | < .001 |  |
| HHA+LASER  & |  | ctr |  | -35.333 |  | 1.119 |  | -31.584 |  | < .001 |  |
|  | | | | | | | | | | | |
| *Note.*  P-value adjusted for comparing a family of 6 | | | | | | | | | | | |

**Figure 3C: Gene expression of TNF-α at 48 h**

| **Post Hoc Comparisons - V1** | | | | | | | | | | | |
| --- | --- | --- | --- | --- | --- | --- | --- | --- | --- | --- | --- |
|  | |  | | **Mean Difference** | | **SE** | | **t** | | **p _tukey_** | |
| CTR+LASER |  | HCC |  | 12.767 |  | 2.268 |  | 5.628 |  | 0.001 |  |
| * |  | HCC+LASER |  | -3.300 |  | 2.268 |  | -1.455 |  | 0.696 |  |
|  |  | HHA |  | 9.933 |  | 2.268 |  | 4.379 |  | 0.009 |  |
|  |  | HHA+LASER |  | 26.800 |  | 2.268 |  | 11.815 |  | < .001 |  |
|  |  | ctr |  | 34.700 |  | 2.268 |  | 15.298 |  | < .001 |  |
| HCC |  | HCC+LASER |  | -16.067 |  | 2.268 |  | -7.083 |  | < .001 |  |
| § |  | HHA |  | -2.833 |  | 2.268 |  | -1.249 |  | 0.805 |  |
|  |  | HHA+LASER |  | 14.033 |  | 2.268 |  | 6.187 |  | < .001 |  |
|  |  | ctr |  | 21.933 |  | 2.268 |  | 9.670 |  | < .001 |  |
| HCC+LASER |  | HHA |  | 13.233 |  | 2.268 |  | 5.834 |  | < .001 |  |
| ° |  | HHA+LASER |  | 30.100 |  | 2.268 |  | 13.270 |  | < .001 |  |
|  |  | ctr |  | 38.000 |  | 2.268 |  | 16.753 |  | < .001 |  |
| HHA |  | HHA+LASER |  | 16.867 |  | 2.268 |  | 7.436 |  | < .001 |  |
| $ |  | ctr |  | 24.767 |  | 2.268 |  | 10.919 |  | < .001 |  |
| HHA+LASER  & |  | ctr |  | 7.900 |  | 2.268 |  | 3.483 |  | 0.041 |  |
|  | | | | | | | | | | | |
| *Note.*  P-value adjusted for comparing a family of 6 | | | | | | | | | | | |

**Figure 3D: Gene expression of TGF-β at 24 h**

| **Post Hoc Comparisons - V1** | | | | | | | | | | | | | | |  |
| --- | --- | --- | --- | --- | --- | --- | --- | --- | --- | --- | --- | --- | --- | --- | --- |
|  |  | | **Mean Difference** | | | | **SE** | | **t** | | | **p _tukey_** | | |  |
| CTR+LASER |  | HCC | |  | 10.200 |  | | 2.645 | |  | 3.856 | |  | 0.022 |  |
| * |  | HCC+LASER | |  | 0.467 |  | | 2.645 | |  | 0.176 | |  | 1.000 |  |
|  |  | HHA | |  | -72.033 |  | | 2.645 | |  | -27.229 | |  | < .001 |  |
|  |  | HHA+LASER | |  | 35.100 |  | | 2.645 | |  | 13.268 | |  | < .001 |  |
|  |  | ctr | |  | 45.200 |  | | 2.645 | |  | 17.086 | |  | < .001 |  |
| HCC |  | HCC+LASER | |  | -9.733 |  | | 2.645 | |  | -3.679 | |  | 0.029 |  |
| § |  | HHA | |  | -82.233 |  | | 2.645 | |  | -31.085 | |  | < .001 |  |
|  |  | HHA+LASER | |  | 24.900 |  | | 2.645 | |  | 9.412 | |  | < .001 |  |
|  |  | ctr | |  | 35.000 |  | | 2.645 | |  | 13.230 | |  | < .001 |  |
| HCC+LASER |  | HHA | |  | -72.500 |  | | 2.645 | |  | -27.405 | |  | < .001 |  |
| ° |  | HHA+LASER | |  | 34.633 |  | | 2.645 | |  | 13.092 | |  | < .001 |  |
|  |  | ctr | |  | 44.733 |  | | 2.645 | |  | 16.909 | |  | < .001 |  |
| HHA |  | HHA+LASER | |  | 107.133 |  | | 2.645 | |  | 40.497 | |  | < .001 |  |
| $ |  | ctr | |  | 117.233 |  | | 2.645 | |  | 44.315 | |  | < .001 |  |
| HHA+LASER  & |  | ctr | |  | 10.100 |  | | 2.645 | |  | 3.818 | |  | 0.023 |  |
|  | | | | | | | | | | | | | | |  |
| *Note.*  P-value adjusted for comparing a family of 6 | | | | | | | | | | | | | | |  |

**Figure 3D: Gene expression of TGF-β at 48 h**

| **Post Hoc Comparisons - V1** | | | | | | | | | | | |
| --- | --- | --- | --- | --- | --- | --- | --- | --- | --- | --- | --- |
|  | |  | | **Mean Difference** | | **SE** | | **t** | | **p _tukey_** | |
| CTR+LASER |  | HCC |  | -14.333 |  | 1.270 |  | -11.283 |  | < .001 |  |
| * |  | HCC+LASER |  | -9.733 |  | 1.270 |  | -7.662 |  | < .001 |  |
|  |  | HHA |  | 35.433 |  | 1.270 |  | 27.893 |  | < .001 |  |
|  |  | HHA+LASER |  | 30.333 |  | 1.270 |  | 23.879 |  | < .001 |  |
|  |  | ctr |  | 60.433 |  | 1.270 |  | 47.573 |  | < .001 |  |
| HCC |  | HCC+LASER |  | 4.600 |  | 1.270 |  | 3.621 |  | 0.032 |  |
| § |  | HHA |  | 49.767 |  | 1.270 |  | 39.177 |  | < .001 |  |
|  |  | HHA+LASER |  | 44.667 |  | 1.270 |  | 35.162 |  | < .001 |  |
|  |  | ctr |  | 74.767 |  | 1.270 |  | 58.857 |  | < .001 |  |
| HCC+LASER |  | HHA |  | 45.167 |  | 1.270 |  | 35.555 |  | < .001 |  |
| ° |  | HHA+LASER |  | 40.067 |  | 1.270 |  | 31.541 |  | < .001 |  |
|  |  | ctr |  | 70.167 |  | 1.270 |  | 55.236 |  | < .001 |  |
| HHA |  | HHA+LASER |  | -5.100 |  | 1.270 |  | -4.015 |  | 0.017 |  |
| $ |  | ctr |  | 25.000 |  | 1.270 |  | 19.680 |  | < .001 |  |
| HHA+LASER  & |  | ctr |  | 30.100 |  | 1.270 |  | 23.695 |  | < .001 |  |
|  | | | | | | | | | | | |
| *Note.*  P-value adjusted for comparing a family of 6 | | | | | | | | | | | |

**Figure 4A : Gene expression of AQP3 at 24 h**

| **Post Hoc Comparisons - V1** | | | | | | | | | | | |
| --- | --- | --- | --- | --- | --- | --- | --- | --- | --- | --- | --- |
|  | |  | | **Mean Difference** | | **SE** | | **t** | | **p _tukey_** | |
| CTR+LASER |  | HCC |  | -15.700 |  | 1.117 |  | -14.051 |  | < .001 |  |
| * |  | HCC+LASER |  | -8.333 |  | 1.117 |  | -7.458 |  | < .001 |  |
|  |  | HHA |  | 36.100 |  | 1.117 |  | 32.308 |  | < .001 |  |
|  |  | HHA+LASER |  | 31.367 |  | 1.117 |  | 28.072 |  | < .001 |  |
|  |  | ctr |  | 62.100 |  | 1.117 |  | 55.577 |  | < .001 |  |
| HCC |  | HCC+LASER |  | 7.367 |  | 1.117 |  | 6.593 |  | < .001 |  |
| § |  | HHA |  | 51.800 |  | 1.117 |  | 46.359 |  | < .001 |  |
|  |  | HHA+LASER |  | 47.067 |  | 1.117 |  | 42.123 |  | < .001 |  |
|  |  | ctr |  | 77.800 |  | 1.117 |  | 69.628 |  | < .001 |  |
| HCC+LASER |  | HHA |  | 44.433 |  | 1.117 |  | 39.766 |  | < .001 |  |
| ° |  | HHA+LASER |  | 39.700 |  | 1.117 |  | 35.530 |  | < .001 |  |
|  |  | ctr |  | 70.433 |  | 1.117 |  | 63.035 |  | < .001 |  |
| HHA |  | HHA+LASER |  | -4.733 |  | 1.117 |  | -4.236 |  | 0.011 |  |
| $ |  | ctr |  | 26.000 |  | 1.117 |  | 23.269 |  | < .001 |  |
| HHA+LASER  & |  | ctr |  | 30.733 |  | 1.117 |  | 27.505 |  | < .001 |  |
|  | | | | | | | | | | | |
| *Note.*  P-value adjusted for comparing a family of 6 | | | | | | | | | | | |

**Figure 4A : Gene expression of AQP3 at 48 h**

| **Post Hoc Comparisons - V1** | | | | | | | | | | | | | | | |  |
| --- | --- | --- | --- | --- | --- | --- | --- | --- | --- | --- | --- | --- | --- | --- | --- | --- |
|  |  | | | **Mean Difference** | | | **SE** | | | **t** | | | **p _tukey_** | | |  |
| CTR |  | CTR+LASER |  | | -0.242 |  | | 0.011 |  | | -22.092 |  | | < .001 |  | |
| # |  | HCC |  | | -0.115 |  | | 0.011 |  | | -10.520 |  | | < .001 |  | |
|  |  | HCC+LASER |  | | -0.370 |  | | 0.011 |  | | -33.814 |  | | < .001 |  | |
|  |  | HHA |  | | -0.075 |  | | 0.011 |  | | -6.818 |  | | 0.004 |  | |
|  |  | HHA+LASER |  | | -0.306 |  | | 0.011 |  | | -27.906 |  | | < .001 |  | |
| CTR+LASER |  | HCC |  | | 0.127 |  | | 0.011 |  | | 11.572 |  | | < .001 |  | |
| * |  | HCC+LASER |  | | -0.128 |  | | 0.011 |  | | -11.722 |  | | < .001 |  | |
|  |  | HHA |  | | 0.167 |  | | 0.011 |  | | 15.274 |  | | < .001 |  | |
|  |  | HHA+LASER |  | | -0.064 |  | | 0.011 |  | | -5.814 |  | | 0.009 |  | |
| HCC |  | HCC+LASER |  | | -0.255 |  | | 0.011 |  | | -23.294 |  | | < .001 |  | |
| § |  | HHA |  | | 0.041 |  | | 0.011 |  | | 3.702 |  | | 0.067 |  | |
|  |  | HHA+LASER |  | | -0.190 |  | | 0.011 |  | | -17.386 |  | | < .001 |  | |
| HCC+LASER  ° |  | HHA |  | | 0.296 |  | | 0.011 |  | | 26.996 |  | | < .001 |  | |
|  |  | HHA+LASER |  | | 0.065 |  | | 0.011 |  | | 5.908 |  | | 0.008 |  | |
| HHA  $ |  | HHA+LASER |  | | -0.231 |  | | 0.011 |  | | -21.088 |  | | < .001 |  | |
|  | | | | | | | | | | | | | | | |  |
| *Note.*  P-value adjusted for comparing a family of 6 | | | | | | | | | | | | | | | |  |

**Figure 4B : Western blotting of AQP3**

| **Post Hoc Comparisons - V1** | | | | | | | | | | |  |
| --- | --- | --- | --- | --- | --- | --- | --- | --- | --- | --- | --- |
|  | |  | | **Mean Difference** | | **SE** | | **t** | **p _tukey_** | |  |
| CTR+LASER |  | HCC |  | 23.600 |  | 1.184 |  | 19.926 |  | < .001 |  |
| * |  | HCC+LASER |  | -45.100 |  | 1.184 |  | -38.078 |  | < .001 |  |
|  |  | HHA |  | 22.700 |  | 1.324 |  | 17.142 |  | < .001 |  |
|  |  | HHA+LASER |  | -7.700 |  | 1.184 |  | -6.501 |  | < .001 |  |
|  |  | ctr |  | 24.867 |  | 1.184 |  | 20.995 |  | < .001 |  |
| HCC |  | HCC+LASER |  | -68.700 |  | 1.184 |  | -58.004 |  | < .001 |  |
| § |  | HHA |  | -0.900 |  | 1.324 |  | -0.680 |  | 0.981 |  |
|  |  | HHA+LASER |  | -31.300 |  | 1.184 |  | -26.427 |  | < .001 |  |
|  |  | ctr |  | 1.267 |  | 1.184 |  | 1.069 |  | 0.884 |  |
| HCC+LASER |  | HHA |  | 67.800 |  | 1.324 |  | 51.200 |  | < .001 |  |
| ° |  | HHA+LASER |  | 37.400 |  | 1.184 |  | 31.577 |  | < .001 |  |
|  |  | ctr |  | 69.967 |  | 1.184 |  | 59.073 |  | < .001 |  |
| HHA  $ |  | HHA+LASER |  | -30.400 |  | 1.324 |  | -22.957 |  | < .001 |  |
|  |  | ctr |  | 2.167 |  | 1.324 |  | 1.636 |  | 0.594 |  |
| HHA+LASER  & |  | ctr |  | 32.567 |  | 1.184 |  | 27.496 |  | < .001 |  |
|  | | | | | | | | | | |  |
| *Note.*  P-value adjusted for comparing a family of 6 | | | | | | | | | | |  |

**Figure 5A: Gene expression of integrin αV at 24 h**

| **Post Hoc Comparisons - V1** | | | | | | | | | | |  |
| --- | --- | --- | --- | --- | --- | --- | --- | --- | --- | --- | --- |
|  | |  | | **Mean Difference** | | **SE** | | **t** | | **p _tukey_** |  |
| CTR+LASER |  | HCC |  | -49.967 |  | 1.035 |  | -48.264 |  | < .001 |  |
| * |  | HCC+LASER |  | -42.600 |  | 1.035 |  | -41.148 |  | < .001 |  |
|  |  | HHA |  | 27.983 |  | 1.157 |  | 24.176 |  | < .001 |  |
|  |  | HHA+LASER |  | -4.267 |  | 1.035 |  | -4.121 |  | 0.016 |  |
|  |  | ctr |  | 27.800 |  | 1.035 |  | 26.852 |  | < .001 |  |
| HCC |  | HCC+LASER |  | 7.367 |  | 1.035 |  | 7.116 |  | < .001 |  |
| § |  | HHA |  | 77.950 |  | 1.157 |  | 67.344 |  | < .001 |  |
|  |  | HHA+LASER |  | 45.700 |  | 1.035 |  | 44.142 |  | < .001 |  |
|  |  | ctr |  | 77.767 |  | 1.035 |  | 75.116 |  | < .001 |  |
| HCC+LASER |  | HHA |  | 70.583 |  | 1.157 |  | 60.980 |  | < .001 |  |
| ° |  | HHA+LASER |  | 38.333 |  | 1.035 |  | 37.027 |  | < .001 |  |
|  |  | ctr |  | 70.400 |  | 1.035 |  | 68.000 |  | < .001 |  |
| HHA  $ |  | HHA+LASER |  | -32.250 |  | 1.157 |  | -27.862 |  | < .001 |  |
|  |  | ctr |  | -0.183 |  | 1.157 |  | -0.158 |  | 1.000 |  |
| HHA+LASER  & |  | ctr |  | 32.067 |  | 1.035 |  | 30.974 |  | < .001 |  |
|  | | | | | | | | | | |  |
| *Note.*  P-value adjusted for comparing a family of 6 | | | | | | | | | | |  |

**Figure 5A : Gene expression of integrin αV at 48 h**

| **Post Hoc Comparisons - V1** | | | | | | | | | | | | | | | | |  |
| --- | --- | --- | --- | --- | --- | --- | --- | --- | --- | --- | --- | --- | --- | --- | --- | --- | --- |
|  | |  | | | **Mean Difference** | | | **SE** | | | **t** | | | **p _tukey_** | | |  |
| CTR |  | | CTR+LASER |  | | -0.416 |  | | 0.013 |  | | -32.595 |  | | < .001 |  | |
| # |  | | HCC |  | | -0.680 |  | | 0.013 |  | | -53.277 |  | | < .001 |  | |
|  |  | | HCC+LASER |  | | -1.400 |  | | 0.013 |  | | -109.702 |  | | < .001 |  | |
|  |  | | HHA |  | | -0.326 |  | | 0.013 |  | | -25.534 |  | | < .001 |  | |
|  |  | | HHA+LASER |  | | -0.500 |  | | 0.013 |  | | -39.204 |  | | < .001 |  | |
| CTR+LASER |  | | HCC |  | | -0.264 |  | | 0.013 |  | | -20.682 |  | | < .001 |  | |
| * |  | | HCC+LASER |  | | -0.984 |  | | 0.013 |  | | -77.107 |  | | < .001 |  | |
|  |  | | HHA |  | | 0.090 |  | | 0.013 |  | | 7.061 |  | | 0.003 |  | |
|  |  | | HHA+LASER |  | | -0.084 |  | | 0.013 |  | | -6.608 |  | | 0.004 |  | |
| HCC |  | | HCC+LASER |  | | -0.720 |  | | 0.013 |  | | -56.425 |  | | < .001 |  | |
| § |  | | HHA |  | | 0.354 |  | | 0.013 |  | | 27.743 |  | | < .001 |  | |
|  |  | | HHA+LASER |  | | 0.180 |  | | 0.013 |  | | 14.074 |  | | < .001 |  | |
| HCC+LASER |  | | HHA |  | | 1.074 |  | | 0.013 |  | | 84.168 |  | | < .001 |  | |
| ° |  | | HHA+LASER |  | | 0.900 |  | | 0.013 |  | | 70.499 |  | | < .001 |  | |
| HHA  $ |  | | HHA+LASER |  | | -0.174 |  | | 0.013 |  | | -13.669 |  | | < .001 |  | |
|  | | | | | | | | | | | | | | | | |  |
| *Note.*  P-value adjusted for comparing a family of 6 | | | | | | | | | | | | | | | | |  |

**Figure 5B : Western blotting of integrin αV**

| **Post Hoc Comparisons - V1** | | | | | | | | | | | | | | | | |  |
| --- | --- | --- | --- | --- | --- | --- | --- | --- | --- | --- | --- | --- | --- | --- | --- | --- | --- |
|  | |  | | | **Mean Difference** | | | **SE** | | | **t** | | | **p _tukey_** | | |  |
| CTR+LASER |  | | HCC |  | | 30.533 |  | | 3.690 |  | | 8.275 |  | | < .001 |  | |
| * |  | | HCC+LASER |  | | -6.567 |  | | 3.690 |  | | -1.780 |  | | 0.511 |  | |
|  |  | | HHA |  | | 39.000 |  | | 3.690 |  | | 10.570 |  | | < .001 |  | |
|  |  | | HHA+LASER |  | | 10.867 |  | | 3.690 |  | | 2.945 |  | | 0.099 |  | |
|  |  | | ctr |  | | 46.700 |  | | 3.690 |  | | 12.657 |  | | < .001 |  | |
| HCC |  | | HCC+LASER |  | | -37.100 |  | | 3.690 |  | | -10.055 |  | | < .001 |  | |
| § |  | | HHA |  | | 8.467 |  | | 3.690 |  | | 2.295 |  | | 0.267 |  | |
|  |  | | HHA+LASER |  | | -19.667 |  | | 3.690 |  | | -5.330 |  | | 0.002 |  | |
|  |  | | ctr |  | | 16.167 |  | | 3.690 |  | | 4.382 |  | | 0.009 |  | |
| HCC+LASER |  | | HHA |  | | 45.567 |  | | 3.690 |  | | 12.350 |  | | < .001 |  | |
| ° |  | | HHA+LASER |  | | 17.433 |  | | 3.690 |  | | 4.725 |  | | 0.005 |  | |
|  |  | | ctr |  | | 53.267 |  | | 3.690 |  | | 14.437 |  | | < .001 |  | |
| HHA |  | | HHA+LASER |  | | -28.133 |  | | 3.690 |  | | -7.625 |  | | < .001 |  | |
| $ |  | | ctr |  | | 7.700 |  | | 3.690 |  | | 2.087 |  | | 0.354 |  | |
| HHA+LASER  & |  | | ctr |  | | 35.833 |  | | 3.690 |  | | 9.712 |  | | < .001 |  | |
|  | | | | | | | | | | | | | | | | |  |
| *Note.*  P-value adjusted for comparing a family of 6 | | | | | | | | | | | | | | | | |  |

**Figure 5C : Gene expression of integrin β3 at 24 h**

| **Post Hoc Comparisons - V1** | | | | | | | | | | | |  |  |
| --- | --- | --- | --- | --- | --- | --- | --- | --- | --- | --- | --- | --- | --- |
|  |  | | **Mean Difference** | | **SE** | | **t** | | **p _tukey_** | | |  |  |
| CTR+LASER |  | HCC |  | 38.900 |  | 3.002 |  | 12.957 | |  | < .001 | |  |
| * |  | HCC+LASER |  | -16.133 |  | 3.002 |  | -5.374 | |  | 0.002 | |  |
|  |  | HHA |  | 48.100 |  | 3.002 |  | 16.021 | |  | < .001 | |  |
|  |  | HHA+LASER |  | 16.967 |  | 3.002 |  | 5.651 | |  | 0.001 | |  |
|  |  | ctr |  | 59.833 |  | 3.002 |  | 19.929 | |  | < .001 | |  |
| HCC |  | HCC+LASER |  | -55.033 |  | 3.002 |  | -18.330 | |  | < .001 | |  |
| § |  | HHA |  | 9.200 |  | 3.002 |  | 3.064 | |  | 0.082 | |  |
|  |  | HHA+LASER |  | -21.933 |  | 3.002 |  | -7.305 | |  | < .001 | |  |
|  |  | ctr |  | 20.933 |  | 3.002 |  | 6.972 | |  | < .001 | |  |
| HCC+LASER |  | HHA |  | 64.233 |  | 3.002 |  | 21.394 | |  | < .001 | |  |
| ° |  | HHA+LASER |  | 33.100 |  | 3.002 |  | 11.025 | |  | < .001 | |  |
|  |  | ctr |  | 75.967 |  | 3.002 |  | 25.302 | |  | < .001 | |  |
| HHA |  | HHA+LASER |  | -31.133 |  | 3.002 |  | -10.370 | |  | < .001 | |  |
| $ |  | ctr |  | 11.733 |  | 3.002 |  | 3.908 | |  | 0.020 | |  |
| HHA+LASER  & |  | ctr |  | 42.867 |  | 3.002 |  | 14.278 | |  | < .001 | |  |
|  | | | | | | | | | | | |  |  |
| *Note.*  P-value adjusted for comparing a family of 6 | | | | | | | | | | | |  |  |

**Figure 5C : Gene expression of integrin β3 at 48 h**

| **Post Hoc Comparisons - V1** | | | | | | | | | | | |
| --- | --- | --- | --- | --- | --- | --- | --- | --- | --- | --- | --- |
|  | |  | | **Mean Difference** | | **SE** | | **t** | | **p _tukey_** | |
| CTR |  | CTR+LASER |  | -0.835 |  | 0.009 |  | -89.392 |  | < .001 |  |
| # |  | HCC |  | -0.374 |  | 0.009 |  | -40.047 |  | < .001 |  |
|  |  | HCC+LASER |  | -1.297 |  | 0.009 |  | -138.820 |  | < .001 |  |
|  |  | HHA |  | -0.254 |  | 0.009 |  | -27.180 |  | < .001 |  |
|  |  | HHA+LASER |  | -1.120 |  | 0.009 |  | -119.835 |  | < .001 |  |
| CTR+LASER |  | HCC |  | 0.461 |  | 0.009 |  | 49.345 |  | < .001 |  |
| * |  | HCC+LASER |  | -0.462 |  | 0.009 |  | -49.428 |  | < .001 |  |
|  |  | HHA |  | 0.581 |  | 0.009 |  | 62.212 |  | < .001 |  |
|  |  | HHA+LASER |  | -0.284 |  | 0.009 |  | -30.443 |  | < .001 |  |
| HCC |  | HCC+LASER |  | -0.923 |  | 0.009 |  | -98.773 |  | < .001 |  |
| § |  | HHA |  | 0.120 |  | 0.009 |  | 12.867 |  | < .001 |  |
|  |  | HHA+LASER |  | -0.745 |  | 0.009 |  | -79.788 |  | < .001 |  |
| HCC+LASER |  | HHA |  | 1.043 |  | 0.009 |  | 111.640 |  | < .001 |  |
| ° |  | HHA+LASER |  | 0.177 |  | 0.009 |  | 18.985 |  | < .001 |  |
| HHA  $ |  | HHA+LASER |  | -0.866 |  | 0.009 |  | -92.655 |  | < .001 |  |
|  | | | | | | | | | | | |
| *Note.*  P-value adjusted for comparing a family of 6 | | | | | | | | | | | |

**Figure 5D : Western blotting of integrin β3**
